# Associations between serum metabolites and subclinical atherosclerosis in a Chinese population: the Taizhou Imaging Study

**DOI:** 10.18632/aging.103456

**Published:** 2020-07-09

**Authors:** Yanfeng Jiang, Kexun Zhang, Zhen Zhu, Mei Cui, Yanpeng An, Yingzhe Wang, Chen Suo, Min Fan, Li Jin, Weizhong Tian, Xingdong Chen

**Affiliations:** 1 State Key Laboratory of Genetic Engineering, Human Phenome Institute, and School of Life Sciences, Fudan University, Shanghai, China; 2 Fudan University Taizhou Institute of Health Sciences, Taizhou, Jiangsu, China; 3 Department of Epidemiology, School of Public Health, Fudan University, Shanghai, China; 4 Department of Neurology, Huashan Hospital, Fudan University, Shanghai, China; 5 Metabonomics and Systems Biology Laboratory, School of Life Sciences, Fudan University, Shanghai, China; 6 Taixing Disease Control and Prevention Center, Taizhou, Jiangsu, China; 7 Department of Medical Imaging, Taizhou People’s Hospital Affiliated to Nantong University, Taizhou, Jiangsu, China

**Keywords:** atherosclerosis, metabolomics, metabolites, brachial-ankle pulse wave velocity, carotid intima-media thickness

## Abstract

Metabolomics provides a promising tool for understanding the pathophysiology and identifying biomarkers of atherosclerosis. We aimed to estimate the associations between circulating metabolites and subclinical atherosclerosis in a Chinese cohort. The baseline serum levels of 38 metabolites of 489 individuals were measured using nuclear magnetic resonance. Associations between metabolites and brachial-ankle pulse wave velocity (baPWV) and carotid intima-media thickness (IMT) were determined using a linear regression. A multivariate logistic regression was used to evaluate the associations of metabolites and subclinical atherosclerosis defined as high baPWV (>median) and increased IMT (>median). After adjusting for covariates and multiple testing corrections (false discovery rate; FDR), two branched-chain amino acids (BCAAs; leucine and isoleucine), one ketone (acetoacetate), and two lipids were positively associated with baPWV. Lactate was inversely associated with IMT. Elevated acetoacetate levels (odds ratio: 1.53, 95% confidence interval: 1.20-1.97; FDR <0.001) and four other lipid features were associated with an increased risk of high baPWV. Alterations in circulating lipids and BCAAs were associated with the risk of arterial stiffness in the middle-aged Chinese population. Our findings provide clues to understanding the potential mechanisms of subclinical atherosclerosis; however, further validation in a broader population context and the exploration of potential clinical applications are warranted.

## INTRODUCTION

Atherosclerotic disease, including cardiovascular disease (CVD) and stroke, is the leading cause of disability and mortality globally [1]. Arterial stiffness and carotid intima-media thickness (IMT) are noninvasive ultrasound indicators of subclinical atherosclerosis and are associated with CVD, stroke, and mortality [2–4]. The traditional risk factors for arterial stiffness and increased IMT have been well documented and include aging, smoking, hypertension, diabetes, hyperlipidemia, etc. However, the mechanisms of the pathophysiology and progression of subclinical atherosclerosis are still less understood.

Metabolomics, an emerging technology that can simultaneously detect a wide range of molecular features of metabolism and investigate the perturbed metabolic pathways in the body, provides a promising and unique opportunity to understanding the pathogenesis and identifying biomarkers of diseases [5]. Previous studies have identified key circulating metabolites associated with atherosclerosis and the subsequent risk of atherosclerotic disease [6–8]. Recently, several epidemiological metabolomic studies have focused on the relationships between blood metabolites and subclinical atherosclerosis; however, the results were inconsistent [7, 9–13]. Moreover, all of these studies were conducted in Western countries, and thus, the findings could not be directly generalized to the Asian population because race and lifestyle (e.g., dietary patterns) are critical biological variables associated with the human metabolome [14].

In this study, based on the baseline data of the Taizhou Imaging Study (TIS), we performed an untargeted metabolic analysis to determine the serum metabolites that are associated with arterial stiffness measured by brachial-ankle pulse wave velocity (baPWV), IMT, and subclinical atherosclerosis in a middle-aged Chinese population.

## RESULTS

### Baseline characteristics of the participants

Table 1 shows the selected baseline characteristics of the study population. The average (standard deviation, SD) age of the participants in this study was 59.2 (2.7) years, and 53.8% of them were female. More than one-third of the participants were smokers at baseline, and hyperlipidemia and diabetes were prevalent in nearly half and 12.7% of the study population, respectively. The median (interquartile range, IQR) values of baPWV and IMT were 15.1 (13.7-16.9) m/s and 0.80 (0.70-0.85) mm, respectively.

**Table 1:** Baseline characteristics of the study population.

**Characteristics**	***n***	**Overall**
Total sample size	489	
Age, years, mean ± standard deviation (SD)	489	59.2 ± 2.7
Women, *n* (%)	489	263 (53.8)
Systolic blood pressure, mmHg, mean ± SD	488	139.1 ± 20.0
Diastolic blood pressure, mmHg, mean ± SD	488	81.2 ± 12.4
Body mass index, kg/m^2^, mean ± SD	489	24.2 ± 3.2
Baseline smoking, *n* (%)	486	178 (36.6)
Physical exercise, *n* (%)	489	27 (5.5)
Use of antihypertensive medications, *n* (%)	489	141 (28.8)
Hyperlipidemia, *n* (%)	489	238 (48.7)
Diabetes mellitus, *n* (%)	489	62 (12.7)
Brachial-ankle pulse wave velocity, m/s, median (interquartile range)	468	15.1 (13.7-16.9)
Carotid intima-media thickness, mm, median (interquartile range)	479	0.80 (0.70-0.85)

**Figure 1:**
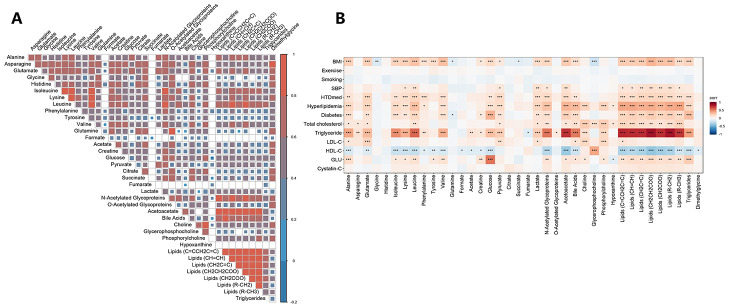
The correlations among concentrations of serum metabolites (**A**) and the correlation matrix between the concentrations of metabolites and cardiovascular disease risk factors (**B**). A partial Spearman correlation analysis was used to calculate the correlation coefficients, adjusting for age and sex. The significance threshold was set at ^*^p<0.05, ^**^p<0.01, and ^***^p<0.001 after correcting for the false discovery rate. Abbreviations: BMI, body mass index; HDL–C, high–density lipoprotein cholesterol; GLU, serum glucose; HTDmed, use of antihypertensive medications; LDL–C, low–density lipoprotein cholesterol; SBP, systolic blood pressure.

We detected 38 metabolic features by untargeted nuclear magnetic resonance (NMR), including amino acids, organic acids, lipids, carbohydrates, choline metabolites, glycoprotein, ketones, bile acid, and chemical intermediates (Supplementary Table 1). Most baseline metabolites were correlated with one another, with the highest correlation coefficient observed among lipids (Figure 1A and Supplementary Table 1). Serum metabolic lipid traits were strongly correlated with routine lipid measurements, including total cholesterol (TC), triglycerides (TGs), high–density lipoprotein cholesterol (HDL–C), and low–density lipoprotein cholesterol (LDL–C) (Figure 1B and Supplementary Table 2). Body mass index (BMI), diabetes, and hyperlipidemia were significantly correlated with most metabolites. Significant correlations were also found between some baseline amino acids and routine lipid traits; for example, leucine was significantly associated with serum TGs (r = 0.58, *p*<0.001).

### Associations between serum metabolites and baPWV and IMT

**Figure 2:**
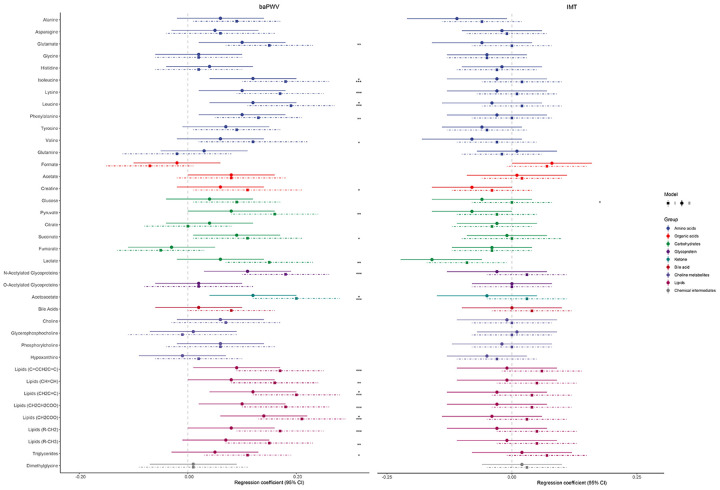
**Linear regression coefficients (95% confidence intervals) between each standard deviation increase in serum metabolites and baPWV (left) and IMT (right).** Model 1 (the dotted lines) was adjusted for age and sex; Model 2 (the solid lines) was additionally adjusted for baseline smoking, physical exercise, body mass index, systolic blood pressure, use of antihypertensive medications, diabetes mellitus, and hyperlipidemia. The significance threshold was set at ^*^p<0.05, ^**^p<0.01, and ^***^p<0.001 after correcting for the false discovery rate. Abbreviations: baPWV, brachial-ankle pulse wave velocity; IMT, carotid intima-media thickness.

Figure 2 and Supplementary Table 3 show the linear regression coefficients and 95% confidence intervals (CIs) for each 1-SD increase in serum metabolites and baPWV and IMT. In the age- and sex-adjusted analyses (Model 1), 20 metabolic features were significantly positively associated with baPWV (false discovery rate [FDR] <0.05; Figure 2 and Supplementary Table 3), and most of them were amino acids and lipids. After adjusting for the covariates in Model 2, five metabolites remained significantly associated with baPWV, including two branched-chain amino acids (BCAAs; leucine and isoleucine), one ketone (acetoacetate), and two lipids. Only lactate was significantly inversely associated with IMT in the fully adjusted model (Model 2: β = -0.16, FDR = 0.025).

### Association between serum metabolites and subclinical atherosclerosis

**Figure 3:**
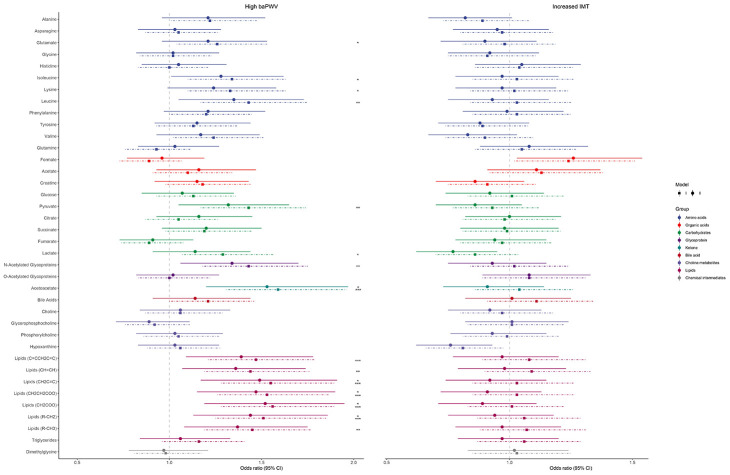
**Associations between serum metabolites and subclinical atherosclerosis.** The point ranges represent odds ratios (95% confidence intervals) between each standard deviation increase in metabolites and high baPWV (left; baPWV >15.1 m/s) and increased IMT (right; IMT >0.80 mm), generated from logistic regression models. Model 1 (the dotted lines) was adjusted for age and sex; Model 2 (the solid lines) was additionally adjusted for baseline smoking, physical exercise, body mass index, systolic blood pressure, use of antihypertensive medications, diabetes mellitus, and hyperlipidemia. The significance threshold was set at ^*^p<0.05, ^**^p<0.01, and ^***^p<0.001 after correcting for the false discovery rate. Abbreviations: baPWV, brachial-ankle pulse wave velocity; IMT, carotid intima-media thickness.

Figure 3 and Supplementary Table 4 show the odds ratios (ORs) and 95% CIs of each metabolite for subclinical atherosclerosis. There was considerable overlap and consistency in the metabolic features between the results of the linear and logistic regressions in terms of baPWV. After adjustment for age and sex, acetoacetate, leucine, isoleucine, and 15 other metabolic signatures were significantly correlated with subclinical atherosclerosis, as indicated by high baPWV (FDR <0.05). In the fully adjusted Model 2, elevated acetoacetate (OR: 1.53, 95% CI: 1.20-1.97; FDR <0.001) and four other lipids were associated with an increased risk of high baPWV. However, no significant associations were found between metabolites and increased IMT in either model.

## DISCUSSION

In this population-based cross-sectional study, we estimated the association between NMR-based metabolomic signatures and the phenotypes of subclinical atherosclerosis. We identified five serum metabolic features independently associated with baPWV, and subclinical atherosclerosis was indicated by high baPWV. These results suggest that perturbations in lipid and BCAA metabolism might be associated with arterial stiffness. Our data provide clues to understanding the potential mechanisms of subclinical atherosclerosis and highlight the potential use of metabolomics for discovering biomarkers for atherosclerosis lipids, fatty acids, and lipoprotein profiles measured by both conventional methods and metabolomic approaches have been confirmed to be associated with CVD, myocardial infarction, and stroke in both cross-sectional and prospective studies [6, 15, 16]. Although the definite mechanism of atherosclerosis continues to be intensively investigated, imbalanced lipid metabolism and its link with inflammation play pivotal roles in the current concepts of atherogenesis [17, 18]. Accumulating evidence has shown associations between lipids and lipoprotein subclasses and subclinical atherosclerosis, although the findings are heterogeneous across studies [7, 9–13]. In this study, we identified four lipids or their moieties that were significantly related to subclinical atherosclerosis, which was in line with previous studies [9, 10, 12, 13]. This relationship was linked to arterial stiffness measured by baPWV. Similarly, several NMR-based fatty acids and small HDL particles were observed to be associated with PWV in the study of Juonala et al. [9]. In addition, lipids were related to PWV in a semirural biracial cohort study (Bogalusa Heart Study) [10]. Moreover, this association was found in studies of males [19] and patients [20]. Similar findings have been reported in terms of the associations between lipids and IMT [7, 13]. However, we found no significant associations between lipids and IMT. For example, using the same NMR platform that was used in this study, Tzoulaki et al. [7] found that several lipids were directly associated with IMT in three cohorts. This inconsistency might be explained by the following reasons. First, the blood metabolic profiles between Chinese individuals and other races differ due to different dietary patterns (the Chinese rural diet is lower in lipids than the Western diet) and genetic backgrounds. Second, the participants in this study were relatively young and had nearly normal lipid levels (e.g., the mean LDL–C was 2.47 mmol/L) and IMT (the median IMT was 0.8 mm), which might attenuate the associations between lipids and IMT. Hou et al. [21] found that normal blood levels of LDL–C and its subclass had lower predictive values for carotid plaque and IMT in young (mean age 48.25 years) general Chinese adults. Third, we only identified eight lipids or their moieties by NMR; therefore, the associations between IMT and other lipids and lipoprotein subclasses detected by mass spectrometry-based platforms or lipidomics should be further investigated. Nevertheless, Juonala and colleagues also observed no evidence of an association between lipids and IMT in adults [9], which is consistent with our findings. Therefore, the associations of lipids with IMT require further investigation and confirmation.

Two metabolites of BCAAs (leucine and isoleucine) were directly associated with baPWV after multivariate adjustment for CVD risk factors and FDR correction. Higher BCAA concentrations have been reported to be associated with the risk of CVD and diabetes [22–24], which highlights their close relationship with atherosclerosis. Previous studies have shown that BCAAs were associated with perturbed lipid metabolism [24, 25]; for example, Wang et al. [25] found that elevated plasma BCAAs were significantly related to a high risk of atherogenic lipids in the Chinese population. We also observed strong correlations between BCAAs and both conventional lipid testing and metabolomic lipid signatures (Figure 1B). Acetoacetic acid can cause damage to the arterial wall by inducing extreme swelling and the loss of mitochondrial cristae in arterial endothelium and myocytes [26]. Here, we highlighted the associations between baPWV and arterial stiffness. Elevated plasma lactate could increase the risk of diabetes [27], showing a positive [7] or no [9] association with IMT in previous studies; however, a negative association was observed in this study, which requires further confirmation.

To the best of our knowledge, this is the first study to estimate the association between circulating metabolites and phenotypes of subclinical atherosclerosis in middle-aged Chinese community residents. This study indicates that the administration of circulating metabolites might be a potential measure for lowering baPWV and a method for the treatment or prevention of atherosclerosis. For example, concentrations of circulating BCAAs often directly reflect the level of dietary consumption, and a BCAA-restricted diet could be used to prevent or delay atherosclerosis. However, several limitations should be acknowledged. First, the cross-sectional design limits the causal inference between metabolites and subclinical atherosclerosis. Perspective studies with repeated phenotype measurements to evaluate the incidence and progression of atherosclerosis and serial blood samples for repeated metabolomic profiling are warranted to validate the relationship and to explore the role of dynamic changes in metabolites involved in atherogenesis. Second, we were unable to find available Chinese population-based cohorts that assessed the phenotypes of subclinical atherosclerosis and blood metabolomic features to replicate our preliminary findings. Nevertheless, we used a conservative statistical threshold with FDR correction, and the significant metabolites were similar to those identified in previous Western studies, which suggests the biological relationship between these identified metabolites and subclinical atherosclerosis. Third, the serum metabolites could be affected by age. The participants in our study were relatively young and with a narrow age range, which limits the generalizability of our results. Further studies are warranted to validate our findings in older people or other age groups. Finally, our analyses were limited to untargeted NMR-based metabolic signatures, and we only obtained the relative concentrations of measured metabolites. Mass spectrometry-based analytical platforms or a combination of both approaches are attractive prospects to extend metabolomic information and to measure the absolute concentrations of metabolites in future studies.

## CONCLUSIONS

In summary, we found that alterations in circulating lipids and BCAAs were associated with the risk of arterial stiffness in a middle-aged Chinese population. These findings should be further validated in other populations, and the underlying biological mechanisms should be further explored to investigate their potential clinical applications.

## MATERIALS AND METHODS

### Study design and participants

Data from the participants in this study were extracted from the TIS, which is a subcohort of the Taizhou Longitudinal Study (TZL) [28]. The TIS is an ongoing well-phenotyped community-based neuroimaging cohort that aims to investigate risk factors and understand the pathological process and progression of cerebrovascular diseases and dementia. The study design of the TIS has been detailed previously [29, 30]. In phase I of the TIS, Han Chinese individuals aged 55–65 years old without physician-diagnosed stroke, cardiovascular disease (including coronary heart disease and valvular heart disease), cancer, psychiatric disorders, or severe liver or renal disease from two villages of Taixing were invited to participate in the baseline examination. Experienced clinicians from the Huashan Hospital and the Taizhou People’s Hospital conducted clinical interviews of all participants using their medical records that maintained by them. Moreover, a brain MRI and electrocardiogram were also performed for each participant to detect undiagnosed brain and heart disease.

A total of 624 individuals met the inclusion criteria, and 562 completed the phase I baseline survey between March 2013 and January 2015, with a response rate of 90%. All baseline interviews, clinical examinations, and fasting biospecimen (e.g., blood, urine, faeces) collections were conducted by experienced technicians at Taizhou People’s Hospital on the same day. For the present study, 494 participants with adequate baseline serum samples for metabolomic profiling were included. Among them, 15 and 26 individuals were excluded from the subsequent analysis due to a lack of baPWV and IMT data. Finally, 479 and 468 participants were included in the evaluation of the cross-sectional associations of serum metabolites with baPWV and IMT (Supplementary Figure 1). The TIS was approved by the Ethics Committee of the School of Life Sciences, Fudan University, Shanghai, China (institutional review board approval number: 496), and all included individuals provided their written informed consent.

### Measurements of brachial-ankle pulse wave velocity and carotid intima-media thickness

We have detailed the methods for the measurements of baPWV and IMT in our published literature [29]. Briefly, assessments were conducted in a quiet room at a constant temperature with the participants in the supine position after at least 5–15 minutes of rest by the same well-trained sonographer. baPWV was determined using a fully automatic waveform analyzer (BP–203RPE III; OMRON HEALTHCARE Co., Ltd., Tokyo, Japan), with baPWV calculated as the length of the arterial segment from the brachium to the ankle (estimated from each participant’s body height) divided by the transmission time of the pulse wave. The mean baPWV of bilateral measurements was used for further analysis [7, 9, 10]. IMT was measured approximately 1 cm proximal and distal to the carotid bulb for three cardiac cycles using a color Doppler ultrasound diagnostic scanner (Acuson S2000; Siemens AG, Munich, Germany). The bilateral common and internal carotid IMT were measured, and the mean values of the common carotid IMT were used for the statistical analysis [7, 9]. baPWV and IMT have been demonstrated to be good indicators of the progression of atherosclerosis. We defined high baPWV (baPWV >15.1 m/s (>median)) and increased IMT (IMT >0.80 mm (>median)) as subclinical atherosclerosis [25].

### Baseline serum sample collection and metabolomic analysis

At baseline, 10-mL venous blood samples were collected from each participant by a certificated nurse between 7:00 and 8:00 AM after an overnight fast. A 5 mL blood aliquot without anticoagulant was centrifuged, and 2.5 mL of serum and one blood clot were aliquoted into 0.5-mL barcoded cryogenic tubes and stored at -80 °C in the Biobank of the Fudan University Taizhou Institute of Health Sciences for future use [29, 30]. The serum samples used in the present study were not subjected to a second freeze-thaw cycle prior to the metabolomic analysis.

From June to July 2016, each available baseline serum sample (160 μL) was imbedded in dry ice and transported to the metabonomic platform of the State Key Laboratory of Genetic Engineering, Fudan University for metabolomic analysis. Each serum sample was mixed with 320 μL of phosphate buffer (45 mM, 50% D_2_O, 0.9% NaCl, pH 7.43), placed into a 5-mm NMR tube and underwent untargeted metabolomic profiling using a Bruker AVIII 600 MHz NMR spectrometer (Bruker Biospin, Germany). All the detections were followed using a standardized procedure and were acquired with similar parameters as previous studies [31–33]. For each sample, three one-dimensional spectra were acquired, including the NOESYPR1D sequence, the Carr-Purcell-Meibom-Gill (CPMG) sequence, and the diffusion-edited spectrum. Additionally, a series of two-dimensional spectra were collected from pooled samples for spectral assignment [31–33]. Data quality control and instrument variability were determined by the evaluations of the peak shape and half-peak width of endogenous metabolites, using the chemical shift of α-glucose (a doublet peak at *δ* 5.23) as a reference. The sample was deleted or reassessed if peak type changed or if the half-peak width widened in its detected signals. All spectra were baseline- and phase-corrected manually via TOPSPIN (v3.6.0, Bruker Biospin, Germany). After removing the water (*δ* 4.47-5.17), urea (*δ* 5.60-6.50), and ethanol (*δ* 3.62-3.68, *δ* 1.09-1.21) signals, each spectrum was segmented into an equal bucket of 0.003 ppm within δ 0.50-9.00 using the AMIX software package (v3.9.15, Bruker Biospin, Germany) and was normalized to the volume of the serum samples to represent the relative concentrations of all metabolites [7, 32, 33].

### Assessment of covariates

The baseline characteristics of the participants were collected by a detailed interviewer-administered questionnaire, including demographics (age and sex), lifestyle (smoking and physical exercise), medical and medication histories, etc. [29, 30]. Blood pressure was measured on the right upper arm with the participants in a seated position after at least 10 minutes of rest, and the mean of two measurements taken at a 5-minute interval was recorded and used for the statistical analysis. BMI (unit: kg/m^2^) was calculated as body weight divided by height squared. Overnight fasting serum glucose, TC, TGs, HDL–C, LDL–C, and cystatin C were tested by an automatic biochemical analyzer (TBA–40FR; TOSHIBA Corp., Tokyo, Japan). Baseline hypertension was defined by a systolic blood pressure ≥140 mmHg or a diastolic blood pressure ≥90 mmHg, a previous diagnosis of hypertension, or the use of antihypertensive drugs. Diabetes mellitus was defined as previously diagnosed diabetes, serum glucose levels ≥7.0 mmol/L, or the use of antidiabetic agents. Hyperlipidemia was defined as previously diagnosed hyperlipidemia, TC ≥5.2 mmol/L, TGs ≥1.7 mmol/L, or current lipid-lowering treatment.

### Statistical analysis

Continuous variables are presented as the mean (SD) or median (IQR) as appropriate, while categorical variables are expressed as frequencies (%). Metabolite relative concentrations were inversely rank transformed and modeled in standard deviation units (per 1-SD) when possible. A partial Spearman correlation analysis was used to calculate the correlation coefficients among concentrations of metabolites and the correlation matrix between concentrations of metabolites and confounding factors (CVD risk factors), adjusting for age and sex. Linear regression models were used to evaluate the associations of baPWV and IMT (continuous variables were also rank transformed because of their skewed distributions) with each 1-SD increase in individual metabolites. Binary logistic regression models were used to assess the associations of subclinical atherosclerosis (i.e., high baPWV and increased IMT) and each 1-SD increase in the relative concentrations of metabolites. Estimations were adjusted for confounding factors in two successive models: Model 1 was adjusted for age and sex, and Model 2 was additionally adjusted for baseline smoking (yes/no), physical exercise (yes/no), BMI (continuous), systolic blood pressure (continuous), use of antihypertension medications (yes/no), diabetes mellitus (yes/no), and hyperlipidemia (yes/no). The Benjamini-Hochberg correction was used for multiple testing, and a FDR value <0.05 was considered statistically significant. All analyses were performed using R 3.5.2 (R core team).

## Supplementary Material

Supplementary Figure 1

Supplementary Table 1

Supplementary Table 2

Supplementary Table 3

Supplementary Table 4
