# Supplementary material for: Associations between serum metabolites and subclinical atherosclerosis in a Chinese population: the Taizhou Imaging Study
Source: Aging (Albany NY). 2020 Jul 9;12(15):15302–13. doi: 10.18632/aging.103456 (PMC7467377; doi:10.18632/aging.103456)
Supplement: Supplementary Figure 1 [file aging-12-103456-s005..pdf]

SUPPLEMENTARY FIGURE

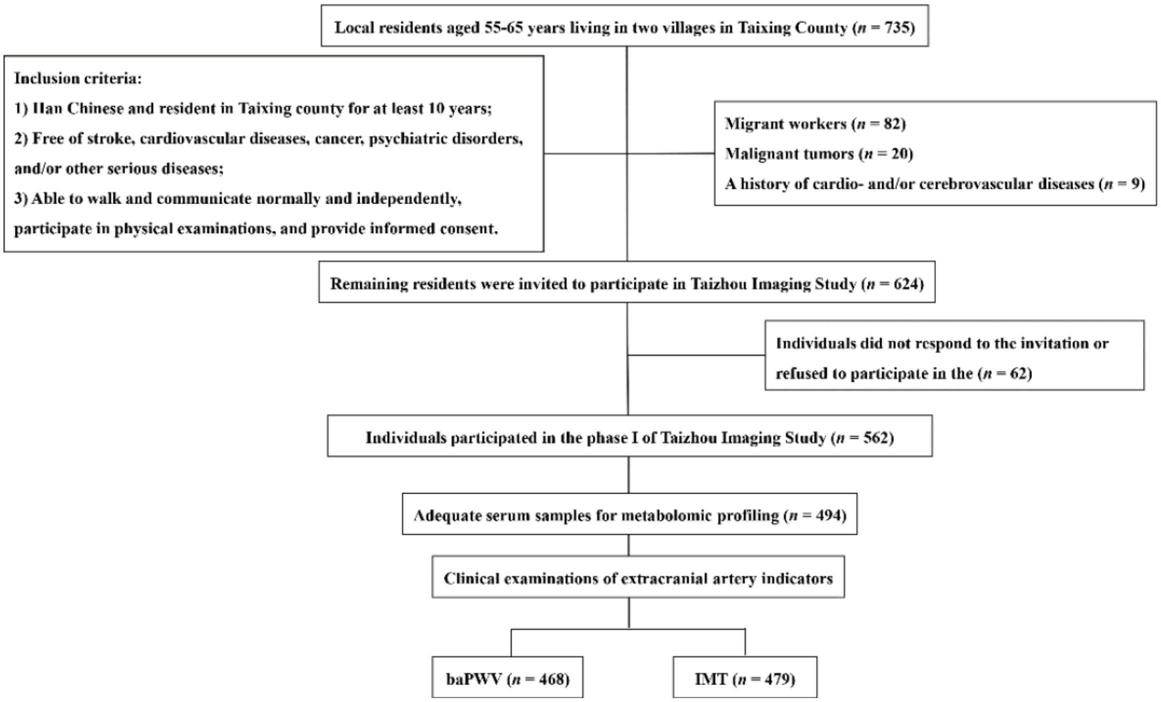

Supplementary Figure 1. Flow chart of study design and participants selection.
