# Supplementary material for: Associations between serum metabolites and subclinical atherosclerosis in a Chinese population: the Taizhou Imaging Study
Source: Aging (Albany NY). 2020 Jul 9;12(15):15302–13. doi: 10.18632/aging.103456 (PMC7467377; doi:10.18632/aging.103456)
Supplement: Supplementary Table 1 [file aging-12-103456-s002..docx]

**Supplementary Table 1. Partial Spearman correlations among serum metabolites in the Taizhou Imaging Study.**

|  | Asparagine | Glutamate | Glycine | Histidine | Isoleucine | Lysine | Leucine | Phenylalanine | Tyrosine |
| --- | --- | --- | --- | --- | --- | --- | --- | --- | --- |
| Alanine | 0.60^***^ | 0.67^***^ | 0.35^***^ | 0.50^***^ | 0.55^***^ | 0.60^***^ | 0.65^***^ | 0.43^***^ | 0.51^***^ |
| Asparagine | 1.00 | 0.68^***^ | 0.63^***^ | 0.77^***^ | 0.56^***^ | 0.71^***^ | 0.64^***^ | 0.50^***^ | 0.45^***^ |
| Glutamate |  | 1.00 | 0.46^***^ | 0.57^***^ | 0.65^***^ | 0.75^***^ | 0.75^***^ | 0.63^***^ | 0.46^***^ |
| Glycine |  |  | 1.00 | 0.59^***^ | 0.37^***^ | 0.44^***^ | 0.34^***^ | 0.43^***^ | 0.32^***^ |
| Histidine |  |  |  | 1.00 | 0.48^***^ | 0.62^***^ | 0.56^***^ | 0.54^***^ | 0.48^***^ |
| Isoleucine |  |  |  |  | 1.00 | 0.81^***^ | 0.85^***^ | 0.50^***^ | 0.38^***^ |
| Lysine |  |  |  |  |  | 1.00 | 0.85^***^ | 0.62^***^ | 0.51^***^ |
| Leucine |  |  |  |  |  |  | 1.00 | 0.54^***^ | 0.45^***^ |
| Phenylalanine |  |  |  |  |  |  |  | 1.00 | 0.54^***^ |
| Tyrosine |  |  |  |  |  |  |  |  | 1.00 |

(Table S1 Continued)

|  | Valine | Glutamine | Formate | Acetate | Creatine | Glucose | Pyruvate | Citrate | Succinate | Fumarate | Lactate |
| --- | --- | --- | --- | --- | --- | --- | --- | --- | --- | --- | --- |
| Alanine | 0.58^***^ | 0.40^***^ | 0.07 | 0.40^***^ | 0.46^***^ | 0.54^***^ | 0.61^***^ | 0.44^***^ | 0.34^***^ | 0.04 | 0.56^***^ |
| Asparagine | 0.61^***^ | 0.72^***^ | 0.16^***^ | 0.65^***^ | 0.65^***^ | 0.59^***^ | 0.40^***^ | 0.75^***^ | 0.74^***^ | 0.04 | 0.40^***^ |
| Glutamate | 0.69^***^ | 0.39^***^ | 0.12^*^ | 0.59^***^ | 0.63^***^ | 0.50^***^ | 0.67^***^ | 0.49^***^ | 0.59^***^ | 0.02 | 0.71^***^ |
| Glycine | 0.35^***^ | 0.62^***^ | 0.06 | 0.44^***^ | 0.55^***^ | 0.34^***^ | 0.24^***^ | 0.53^***^ | 0.59^***^ | 0.00 | 0.25^***^ |
| Histidine | 0.57^***^ | 0.72^***^ | 0.26^***^ | 0.64^***^ | 0.61^***^ | 0.53^***^ | 0.30^***^ | 0.71^***^ | 0.64^***^ | 0.08 | 0.31^***^ |
| Isoleucine | 0.82^***^ | 0.34^***^ | 0.04 | 0.56^***^ | 0.53^***^ | 0.52^***^ | 0.41^***^ | 0.37^***^ | 0.49^***^ | -0.06 | 0.37^***^ |
| Lysine | 0.85^***^ | 0.49^***^ | 0.05 | 0.76^***^ | 0.66^***^ | 0.55^***^ | 0.39^***^ | 0.50^***^ | 0.63^***^ | 0.01 | 0.45^***^ |
| Leucine | 0.88^***^ | 0.35^***^ | 0.06 | 0.59^***^ | 0.56^***^ | 0.59^***^ | 0.45^***^ | 0.43^***^ | 0.54^***^ | 0.02 | 0.48^***^ |
| Phenylalanine | 0.53^***^ | 0.39^***^ | 0.17^***^ | 0.53^***^ | 0.54^***^ | 0.30^***^ | 0.44^***^ | 0.36^***^ | 0.52^***^ | 0.07 | 0.48^***^ |
| Tyrosine | 0.54^***^ | 0.44^***^ | 0.23^***^ | 0.43^***^ | 0.47^***^ | 0.43^***^ | 0.24^***^ | 0.33^***^ | 0.31^***^ | 0.10^*^ | 0.22^***^ |
| Valine | 1.00 | 0.39^***^ | 0.10^*^ | 0.61^***^ | 0.59^***^ | 0.56^***^ | 0.38^***^ | 0.44^***^ | 0.50^***^ | 0.03 | 0.40^***^ |
| Glutamine |  | 1.00 | 0.23^***^ | 0.61^***^ | 0.47^***^ | 0.49^***^ | 0.16^***^ | 0.71^***^ | 0.66^***^ | 0.01 | 0.07 |
| Formate |  |  | 1.00 | 0.20^***^ | 0.21^***^ | 0.24^***^ | 0.08 | 0.19^***^ | 0.12^**^ | 0.11^*^ | 0.00 |
| Acetate |  |  |  | 1.00 | 0.56^***^ | 0.47^***^ | 0.26^***^ | 0.55^***^ | 0.63^***^ | 0.01 | 0.23^***^ |
| Creatine |  |  |  |  | 1.00 | 0.55^***^ | 0.39^***^ | 0.46^***^ | 0.54^***^ | -0.03 | 0.41^***^ |
| Glucose |  |  |  |  |  | 1.00 | 0.31^***^ | 0.52^***^ | 0.41^***^ | 0.02 | 0.16^***^ |
| Pyruvate |  |  |  |  |  |  | 1.00 | 0.28^***^ | 0.34^***^ | 0.05 | 0.65^***^ |
| Citrate |  |  |  |  |  |  |  | 1.00 | 0.70^***^ | 0.05 | 0.25^***^ |
| Succinate |  |  |  |  |  |  |  |  | 1.00 | 0.06 | 0.34^***^ |
| Fumarate |  |  |  |  |  |  |  |  |  | 1.00 | 0.07 |
| Lactate |  |  |  |  |  |  |  |  |  |  | 1.00 |

(Table S1 Continued)

|  | *N*-Acetylated Glycoproteins | *O*-Acetylated Glycoproteins | Acetoacetate | Bile Acids | Choline | Glycerophosphocholine | Phosphorylcholine | Hypoxanthine |
| --- | --- | --- | --- | --- | --- | --- | --- | --- |
| Alanine | 0.65^***^ | 0.53^***^ | 0.41^***^ | 0.44^***^ | 0.48^***^ | 0.24^***^ | 0.49^***^ | 0.09^*^ |
| Asparagine | 0.69^***^ | 0.77^***^ | 0.36^***^ | 0.28^***^ | 0.76^***^ | 0.48^***^ | 0.72^***^ | 0.04 |
| Glutamate | 0.77^***^ | 0.54^***^ | 0.49^***^ | 0.35^***^ | 0.58^***^ | 0.23^***^ | 0.56^***^ | 0.18^***^ |
| Glycine | 0.43^***^ | 0.64^***^ | 0.07 | 0.09 | 0.47^***^ | 0.37^***^ | 0.44^***^ | 0.07 |
| Histidine | 0.54^***^ | 0.76^***^ | 0.26^***^ | 0.26^***^ | 0.67^***^ | 0.44^***^ | 0.64^***^ | 0.06 |
| Isoleucine | 0.79^***^ | 0.51^***^ | 0.57^***^ | 0.30^***^ | 0.44^***^ | 0.20^***^ | 0.44^***^ | 0.05 |
| Lysine | 0.75^***^ | 0.65^***^ | 0.51^***^ | 0.22^***^ | 0.57^***^ | 0.19^***^ | 0.51^***^ | 0.09^*^ |
| Leucine | 0.83^***^ | 0.52^***^ | 0.74^***^ | 0.50^***^ | 0.60^***^ | 0.25^***^ | 0.60^***^ | 0.08 |
| Phenylalanine | 0.56^***^ | 0.50^***^ | 0.24^***^ | 0.28^***^ | 0.42^***^ | 0.14^**^ | 0.36^***^ | 0.26^***^ |
| Tyrosine | 0.38^***^ | 0.50^***^ | 0.14^**^ | 0.21^***^ | 0.38^***^ | 0.20^***^ | 0.39^***^ | 0.02 |
| Valine | 0.65^***^ | 0.53^***^ | 0.52^***^ | 0.31^***^ | 0.57^***^ | 0.26^***^ | 0.56^***^ | 0.05 |
| Glutamine | 0.40^***^ | 0.94^***^ | 0.09^*^ | 0.10^*^ | 0.51^***^ | 0.43^***^ | 0.49^***^ | -0.06 |
| Formate | 0.07 | 0.19^***^ | -0.07 | 0.14^**^ | 0.16^***^ | 0.18^***^ | 0.19^***^ | 0.05 |
| Acetate | 0.54^***^ | 0.70^***^ | 0.32^***^ | 0.19^***^ | 0.51^***^ | 0.29^***^ | 0.49^***^ | 0.05 |
| Creatine | 0.64^***^ | 0.55^***^ | 0.25^***^ | 0.24^***^ | 0.55^***^ | 0.29^***^ | 0.53^***^ | 0.14^**^ |
| Glucose | 0.59^***^ | 0.57^***^ | 0.39^***^ | 0.38^***^ | 0.55^***^ | 0.39^***^ | 0.59^***^ | -0.15^**^ |
| Pyruvate | 0.57^***^ | 0.27^***^ | 0.37^***^ | 0.35^***^ | 0.31^***^ | 0.15^***^ | 0.29^***^ | 0.21^***^ |
| Citrate | 0.46^***^ | 0.69^***^ | 0.21^***^ | 0.15^**^ | 0.62^***^ | 0.45^***^ | 0.58^***^ | -0.07 |
| Succinate | 0.57^***^ | 0.70^***^ | 0.38^***^ | 0.19^***^ | 0.62^***^ | 0.40^***^ | 0.54^***^ | 0.11^*^ |
| Fumarate | -0.06 | 0.01 | -0.02 | 0.09^*^ | 0.08 | 0.06 | 0.06 | 0.16^***^ |
| Lactate | 0.51^***^ | 0.20^***^ | 0.35^***^ | 0.32^***^ | 0.35^***^ | 0.09^*^ | 0.32^***^ | 0.40^***^ |
| *N*-Acetylated Glycoproteins | 1.00 | 0.56^***^ | 0.66^***^ | 0.52^***^ | 0.56^***^ | 0.22^***^ | 0.54^***^ | 0.12^**^ |
| *O*-Acetylated Glycoproteins |  | 1.00 | 0.22^***^ | 0.20^***^ | 0.55^***^ | 0.44^***^ | 0.54^***^ | -0.02 |
| Acetoacetate |  |  | 1.00 | 0.61^***^ | 0.40^***^ | 0.05 | 0.39^***^ | 0.06 |
| Bile Acids |  |  |  | 1.00 | 0.40^***^ | 0.27^***^ | 0.46^***^ | 0.11^*^ |
| Choline |  |  |  |  | 1.00 | 0.56^***^ | 0.93^***^ | 0.12^*^ |
| Glycerophosphocholine |  |  |  |  |  | 1.00 | 0.69^***^ | -0.07 |
| Phosphorylcholine |  |  |  |  |  |  | 1.00 | 0.06 |
| Hypoxanthine |  |  |  |  |  |  |  | 1.00 |

(Table S1 Continued)

|  | Lipids  (C=CC*H2*C=C) | Lipids  (C*H*=CH) | Lipids  (C*H2*C=C) | Lipids  (C*H2*CH2COO) | Lipids  (C*H2*COO) | Lipids  (R-C*H2*) | Lipids  (R-C*H3*) | Triglycerides | Dimethylglycine |
| --- | --- | --- | --- | --- | --- | --- | --- | --- | --- |
| Alanine | 0.59^***^ | 0.55^***^ | 0.61^***^ | 0.56^***^ | 0.57^***^ | 0.54^***^ | 0.54^***^ | 0.38^***^ | 0.51^***^ |
| Asparagine | 0.49^***^ | 0.39^***^ | 0.54^***^ | 0.38^***^ | 0.52^***^ | 0.35^***^ | 0.45^***^ | 0.37^***^ | 0.86^***^ |
| Glutamate | 0.56^***^ | 0.48^***^ | 0.63^***^ | 0.51^***^ | 0.63^***^ | 0.47^***^ | 0.50^***^ | 0.40^***^ | 0.60^***^ |
| Glycine | 0.18^***^ | 0.12^*^ | 0.26^***^ | 0.07 | 0.20^***^ | 0.07 | 0.18^***^ | 0.14^**^ | 0.56^***^ |
| Histidine | 0.37^***^ | 0.32^***^ | 0.40^***^ | 0.25^***^ | 0.39^***^ | 0.24^***^ | 0.35^***^ | 0.32^***^ | 0.68^***^ |
| Isoleucine | 0.60^***^ | 0.54^***^ | 0.73^***^ | 0.59^***^ | 0.70^***^ | 0.56^***^ | 0.56^***^ | 0.47^***^ | 0.47^***^ |
| Lysine | 0.54^***^ | 0.45^***^ | 0.65^***^ | 0.54^***^ | 0.66^***^ | 0.48^***^ | 0.49^***^ | 0.45^***^ | 0.64^***^ |
| Leucine | 0.77^***^ | 0.72^***^ | 0.83^***^ | 0.74^***^ | 0.85^***^ | 0.72^***^ | 0.73^***^ | 0.57^***^ | 0.52^***^ |
| Phenylalanine | 0.33^***^ | 0.29^***^ | 0.41^***^ | 0.24^***^ | 0.37^***^ | 0.23^***^ | 0.29^***^ | 0.32^***^ | 0.47^***^ |
| Tyrosine | 0.26^***^ | 0.24^***^ | 0.29^***^ | 0.21^***^ | 0.28^***^ | 0.19^***^ | 0.23^***^ | 0.29^***^ | 0.47^***^ |
| Valine | 0.54^***^ | 0.50^***^ | 0.61^***^ | 0.53^***^ | 0.66^***^ | 0.50^***^ | 0.52^***^ | 0.48^***^ | 0.53^***^ |
| Glutamine | 0.21^***^ | 0.15^**^ | 0.25^***^ | 0.10^*^ | 0.23^***^ | 0.09^*^ | 0.20^***^ | 0.19^***^ | 0.71^***^ |
| Formate | 0.00 | 0.02 | 0.00 | -0.08 | -0.02 | -0.06 | 0.02 | 0.15^**^ | 0.16^***^ |
| Acetate | 0.37^***^ | 0.29^***^ | 0.42^***^ | 0.30^***^ | 0.43^***^ | 0.26^***^ | 0.33^***^ | 0.32^***^ | 0.64^***^ |
| Creatine | 0.37^***^ | 0.30^***^ | 0.45^***^ | 0.28^***^ | 0.41^***^ | 0.26^***^ | 0.33^***^ | 0.23^***^ | 0.57^***^ |
| Glucose | 0.50^***^ | 0.49^***^ | 0.53^***^ | 0.43^***^ | 0.51^***^ | 0.43^***^ | 0.50^***^ | 0.55^***^ | 0.49^***^ |
| Pyruvate | 0.45^***^ | 0.40^***^ | 0.49^***^ | 0.41^***^ | 0.47^***^ | 0.40^***^ | 0.40^***^ | 0.29^***^ | 0.35^***^ |
| Citrate | 0.30^***^ | 0.22^***^ | 0.32^***^ | 0.19^***^ | 0.33^***^ | 0.18^***^ | 0.30^***^ | 0.25^***^ | 0.69^***^ |
| Succinate | 0.35^***^ | 0.26^***^ | 0.41^***^ | 0.22^***^ | 0.45^***^ | 0.22^***^ | 0.34^***^ | 0.32^***^ | 0.66^***^ |
| Fumarate | -0.02 | 0.03 | -0.05 | -0.07 | -0.03 | -0.05 | 0.00 | 0.27^***^ | 0.04 |
| Lactate | 0.40^***^ | 0.36^***^ | 0.44^***^ | 0.38^***^ | 0.43^***^ | 0.38^***^ | 0.37^***^ | 0.16^***^ | 0.30^***^ |
| *N*-Acetylated Glycoproteins | 0.78^***^ | 0.70^***^ | 0.89^***^ | 0.72^***^ | 0.82^***^ | 0.70^***^ | 0.71^***^ | 0.52^***^ | 0.58^***^ |
| *O*-Acetylated Glycoproteins | 0.35^***^ | 0.29^***^ | 0.41^***^ | 0.25^***^ | 0.37^***^ | 0.23^***^ | 0.33^***^ | 0.30^***^ | 0.75^***^ |
| Acetoacetate | 0.85^***^ | 0.84^***^ | 0.84^***^ | 0.89^***^ | 0.93^***^ | 0.87^***^ | 0.81^***^ | 0.54^***^ | 0.26^***^ |
| Bile Acids | 0.70^***^ | 0.81^***^ | 0.65^***^ | 0.61^***^ | 0.62^***^ | 0.70^***^ | 0.75^***^ | 0.52^***^ | 0.17^***^ |
| Choline | 0.48^***^ | 0.45^***^ | 0.49^***^ | 0.37^***^ | 0.51^***^ | 0.38^***^ | 0.50^***^ | 0.43^***^ | 0.61^***^ |
| Glycerophosphocholine | 0.24^***^ | 0.26^***^ | 0.21^***^ | 0.05 | 0.14^**^ | 0.17^***^ | 0.40^***^ | 0.19^***^ | 0.37^***^ |
| Phosphorylcholine | 0.52^***^ | 0.51^***^ | 0.52^***^ | 0.40^***^ | 0.50^***^ | 0.44^***^ | 0.58^***^ | 0.40^***^ | 0.58^***^ |
| Hypoxanthine | 0.06 | 0.06 | 0.08 | 0.04 | 0.07 | 0.04 | 0.04 | 0.02 | 0.04 |
| Lipids (C=CC*H2*C=C) | 1.00 | 0.96^***^ | 0.94^***^ | 0.93^***^ | 0.91^***^ | 0.95^***^ | 0.94^***^ | 0.57^***^ | 0.37^***^ |
| Lipids (C*H*=CH) |  | 1.00 | 0.91^***^ | 0.90^***^ | 0.87^***^ | 0.96^***^ | 0.96^***^ | 0.62^***^ | 0.28^***^ |
| Lipids (C*H2*C=C) |  |  | 1.00 | 0.92^***^ | 0.93^***^ | 0.93^***^ | 0.91^***^ | 0.59^***^ | 0.42^***^ |
| Lipids (C*H2*CH2COO) |  |  |  | 1.00 | 0.93^***^ | 0.97^***^ | 0.87^***^ | 0.51^***^ | 0.28^***^ |
| Lipids (C*H2*COO) |  |  |  |  | 1.00 | 0.90^***^ | 0.86^***^ | 0.56^***^ | 0.41^***^ |
| Lipids (R-C*H2*) |  |  |  |  |  | 1.00 | 0.95^***^ | 0.53^***^ | 0.25^***^ |
| Lipids (R-C*H3*) |  |  |  |  |  |  | 1.00 | 0.55^***^ | 0.34^***^ |
| Triglycerides |  |  |  |  |  |  |  | 1.00 | 0.27^***^ |
| Dimethylglycine |  |  |  |  |  |  |  |  | 1.00 |

The significance threshold was set at ^*^*P* <0.05, ^**^*P* <0.01, and ^***^*P* <0.001 after false discovery rate correction.
