# Supplementary material for: Associations between serum metabolites and subclinical atherosclerosis in a Chinese population: the Taizhou Imaging Study
Source: Aging (Albany NY). 2020 Jul 9;12(15):15302–13. doi: 10.18632/aging.103456 (PMC7467377; doi:10.18632/aging.103456)
Supplement: Supplementary Table 2 [file aging-12-103456-s001..docx]

**Supplementary Table 2. Partial Spearman correlations between serum metabolites and cardiovascular disease risk factors in the Taizhou Imaging Study.**

| Metabolites | BMI | Exercise | Smoking | SBP | HTDmed | HLP | DM | TC | TG | LDL-C | HDL-C | GLU | CYS-C |
| --- | --- | --- | --- | --- | --- | --- | --- | --- | --- | --- | --- | --- | --- |
| Alanine | 0.24^***^ | 0.04 | -0.08 | 0.09 | 0.14^**^ | 0.21^***^ | 0.17^***^ | 0.11^*^ | 0.39^***^ | 0.12^*^ | -0.17^***^ | 0.19^***^ | 0.04 |
| Asparagine | 0.01 | -0.03 | -0.04 | 0.02 | 0.07 | 0.10 | 0.00 | 0.11^*^ | 0.15^**^ | 0.12 | -0.06 | 0.03 | -0.01 |
| Glutamate | 0.24^***^ | 0.01 | 0.00 | 0.09 | 0.22^***^ | 0.22^***^ | 0.15^**^ | 0.13^*^ | 0.32^***^ | 0.23^***^ | -0.14^**^ | 0.09 | -0.03 |
| Glycine | -0.15^**^ | 0.01 | -0.01 | 0.03 | -0.03 | -0.04 | -0.03 | -0.04 | -0.05 | -0.03 | 0.03 | -0.08 | 0.04 |
| Histidine | 0.03 | 0.00 | -0.10 | -0.03 | 0.07 | -0.01 | -0.02 | -0.01 | 0.07 | 0.05 | -0.07 | -0.04 | -0.01 |
| Isoleucine | 0.23^***^ | -0.02 | -0.04 | 0.09 | 0.15^**^ | 0.20^***^ | 0.17^***^ | 0.04 | 0.47^***^ | -0.03 | -0.22^***^ | 0.11^*^ | 0.07 |
| Lysine | 0.24^***^ | 0.01 | -0.06 | 0.12^*^ | 0.18^***^ | 0.16^***^ | 0.13^*^^*^ | 0.05 | 0.36^***^ | 0.01 | -0.22^***^ | 0.11^*^ | 0.07 |
| Leucine | 0.29^***^ | 0.02 | -0.06 | 0.14^**^ | 0.22^***^ | 0.30^***^ | 0.21^***^ | 0.14^**^ | 0.58^***^ | 0.08 | -0.24^***^ | 0.16^**^ | 0.08 |
| Phenylalanine | 0.16^***^ | -0.02 | -0.05 | 0.02 | 0.17^***^ | 0.12^*^ | 0.08 | 0.07 | 0.14^**^ | 0.07 | -0.11^*^ | -0.03 | 0.05 |
| Tyrosine | 0.19^***^ | 0.07 | -0.11 | 0.03 | 0.10 | 0.01 | 0.05 | -0.05 | 0.08 | 0.00 | -0.14^**^ | 0.11^*^ | 0.02 |
| Valine | 0.30^***^ | 0.03 | -0.07 | 0.10 | 0.17^***^ | 0.17^***^ | 0.16^***^ | 0.06 | 0.36^***^ | 0.02 | -0.17^***^ | 0.15^**^ | 0.03 |
| Glutamine | -0.11^*^ | 0.01 | -0.01 | -0.08 | 0.01 | -0.06 | -0.11^*^ | -0.07 | -0.02 | -0.02 | -0.05 | -0.01 | 0.00 |
| Formate | -0.05 | 0.03 | -0.04 | -0.08 | -0.03 | -0.04 | 0.06 | 0.00 | -0.07 | 0.05 | -0.11^*^ | 0.05 | -0.07 |
| Acetate | -0.02 | 0.00 | -0.01 | -0.07 | 0.08 | 0.03 | -0.01 | 0.04 | 0.14^**^ | 0.08 | -0.13^*^ | -0.02 | 0.05 |
| Creatine | 0.12^*^ | 0.01 | -0.06 | 0.03 | 0.09 | 0.07 | 0.11^*^ | 0.03 | 0.12^*^ | 0.04 | -0.12^*^ | 0.15^**^ | 0.01 |
| Glucose | 0.20^***^ | 0.04 | -0.08 | 0.08 | 0.13^**^ | 0.16^***^ | 0.35^***^ | 0.04 | 0.30^***^ | 0.04 | -0.21^***^ | 0.54^***^ | -0.02 |
| Pyruvate | 0.11^*^ | -0.06 | 0.00 | 0.11^*^ | 0.20^***^ | 0.19^***^ | 0.14^**^ | 0.11^*^ | 0.25^***^ | 0.16^**^ | -0.08 | 0.07 | 0.02 |
| Citrate | -0.09 | 0.00 | -0.03 | -0.07 | -0.03 | 0.01 | -0.04 | 0.06 | 0.01 | 0.08 | 0.02 | 0.01 | 0.11 |
| Succinate | -0.11^*^ | -0.02 | 0.04 | 0.01 | 0.05 | 0.08 | -0.01 | 0.05 | 0.07 | 0.04 | -0.01 | -0.10 | 0.06 |
| Fumarate | -0.09 | 0.01 | -0.07 | -0.07 | 0.01 | 0.06 | 0.01 | 0.08 | -0.12^*^ | -0.01 | 0.10 | 0.00 | 0.01 |
| Lactate | 0.19^***^ | -0.07 | -0.04 | 0.16^**^ | 0.18^***^ | 0.22^***^ | 0.14^**^ | 0.14^*^ | 0.25^***^ | 0.15^*^ | -0.08 | -0.01 | -0.02 |
| *N*-Acetylated Glycoproteins | 0.21^***^ | -0.05 | -0.07 | 0.12^*^ | 0.20^***^ | 0.29^***^ | 0.17^***^ | 0.14^**^ | 0.54^***^ | 0.09 | -0.28^***^ | 0.14^**^ | 0.11 |
| *O*-Acetylated Glycoproteins | -0.03 | 0.02 | -0.06 | -0.01 | 0.08 | 0.02 | -0.05 | -0.03 | 0.10^*^ | 0.00 | -0.09 | 0.00 | 0.01 |
| Acetoacetate | 0.26^***^ | 0.02 | -0.04 | 0.16^**^ | 0.20^***^ | 0.34^***^ | 0.24^***^ | 0.13^*^ | 0.74^***^ | 0.05 | -0.37^***^ | 0.13^*^ | 0.11 |
| Bile Acids | 0.17^***^ | 0.02 | -0.05 | 0.05 | 0.13^**^ | 0.32^***^ | 0.19^***^ | 0.17^**^ | 0.53^***^ | 0.14^*^ | -0.21^***^ | 0.13^*^ | 0.04 |
| Choline | 0.05 | -0.01 | 0.00 | 0.00 | 0.04 | 0.23^***^ | 0.03 | 0.26^***^ | 0.17^***^ | 0.21^***^ | 0.08 | 0.11^*^ | 0.03 |
| Glycerophosphocholine | -0.21^***^ | -0.06 | -0.01 | -0.02 | -0.05 | 0.09 | -0.07 | 0.18^***^ | -0.07 | 0.09 | 0.39^***^ | -0.01 | -0.07 |
| Phosphorylcholine | 0.04 | -0.02 | -0.01 | 0.00 | 0.05 | 0.23^***^ | 0.03 | 0.25^***^ | 0.21^***^ | 0.21^***^ | 0.09 | 0.12^*^ | -0.01 |
| Hypoxanthine | -0.03 | -0.06 | 0.02 | 0.02 | 0.07 | 0.11^*^ | 0.04 | 0.13^*^ | 0.00 | 0.07 | 0.03 | -0.11^*^ | 0.03 |
| Lipids (C=CC*H2*C=C) | 0.26^***^ | 0.00 | -0.06 | 0.13^*^ | 0.20^***^ | 0.39^***^ | 0.22^***^ | 0.22^***^ | 0.77^***^ | 0.13^*^ | -0.31^***^ | 0.15^**^ | 0.10 |
| Lipids (C*H*=CH) | 0.25^***^ | 0.00 | -0.07 | 0.13^*^ | 0.19^***^ | 0.39^***^ | 0.24^***^ | 0.21^***^ | 0.76^***^ | 0.11 | -0.29^***^ | 0.18^**^ | 0.10 |
| Lipids (C*H2*C=C) | 0.26^***^ | -0.01 | -0.07 | 0.16^**^ | 0.22^***^ | 0.36^***^ | 0.22^***^ | 0.18^***^ | 0.76^***^ | 0.07 | -0.32^***^ | 0.17^**^ | 0.13 |
| Lipids (C*H2*CH2COO) | 0.34^***^ | 0.01 | -0.05 | 0.18^**^ | 0.22^***^ | 0.37^***^ | 0.25^***^ | 0.16^**^ | 0.84^***^ | 0.07 | -0.39^***^ | 0.20^***^ | 0.12 |
| Lipids (C*H2*COO) | 0.29^***^ | 0.00 | -0.04 | 0.16^**^ | 0.22^***^ | 0.34^***^ | 0.22^***^ | 0.14^**^ | 0.75^***^ | 0.05 | -0.35^***^ | 0.16^**^ | 0.11 |
| Lipids (R-C*H2*) | 0.29^***^ | 0.00 | -0.06 | 0.16^**^ | 0.20^***^ | 0.38^***^ | 0.24^***^ | 0.18^***^ | 0.83^***^ | 0.07 | -0.32^***^ | 0.17^**^ | 0.12 |
| Lipids (R-C*H3*) | 0.19^***^ | -0.03 | -0.04 | 0.12^*^ | 0.17^***^ | 0.40^***^ | 0.20^***^ | 0.23^***^ | 0.73^***^ | 0.12 | -0.20^***^ | 0.15^**^ | 0.10 |
| Triglycerides | 0.23^***^ | -0.02 | -0.10 | 0.10 | 0.17^***^ | 0.21^***^ | 0.31^***^ | 0.09 | 0.41^***^ | 0.01 | -0.24^***^ | 0.25^***^ | 0.07 |
| Dimethylglycine | 0.01 | 0.00 | 0.00 | -0.01 | 0.07 | 0.03 | -0.06 | 0.04 | 0.09 | 0.09 | -0.12^*^ | 0.02 | 0.04 |

Abbreviations: BMI, body mass index; CYS-C, cystain-C; DM, diabetes mellitus; GLU, glucose; HTDmed, use of antihypertensive medications; HDL–C, high–density lipoprotein cholesterol; HLP, hyperlipidemia; IMT, carotid intima-media thickness; LDL–C, low–density lipoprotein cholesterol; SBP, systolic blood pressure; TC, total cholesterol; TGs, triglycerides. The significance threshold was set at ^*^*P* <0.05, ^**^*P* <0.01, and ^***^*P* <0.001 after false discovery rate correction.
