# Supplementary material for: Associations between serum metabolites and subclinical atherosclerosis in a Chinese population: the Taizhou Imaging Study
Source: Aging (Albany NY). 2020 Jul 9;12(15):15302–13. doi: 10.18632/aging.103456 (PMC7467377; doi:10.18632/aging.103456)
Supplement: Supplementary Table 3 [file aging-12-103456-s004..docx]

**Supplementary Table 3. Linear regression analyses of serum metabolites and baPWV and IMT.**

| Metabolites | Metabolite class | baPWV | | | | | | |  | IMT | | | | | | |
| --- | --- | --- | --- | --- | --- | --- | --- | --- | --- | --- | --- | --- | --- | --- | --- | --- |
|  |  | Model 1 | | |  | Model 2 | | |  | Model 1 | | |  | Model 2 | | |
|  |  | β (SE) | *P* value | FDR |  | β (SE) | *P* value | FDR |  | β (SE) | *P* value | FDR |  | β (SE) | *P* value | FDR |
| Alanine | Amino acids | 0.09 (0.04) | 4.0E-2 | 7.3E-2 |  | 0.06 (0.04) | 1.6E-1 | 2.6E-1 |  | -0.06 (0.04) | 1.5E-1 | 8.8E-1 |  | -0.11 (0.05) | 1.7E-2 | 3.3E-1 |
| Asparagine | Amino acids | 0.06 (0.05) | 1.9E-1 | 2.4E-1 |  | 0.05 (0.04) | 2.5E-1 | 3.6E-1 |  | -0.01 (0.04) | 8.1E-1 | 9.8E-1 |  | -0.02 (0.04) | 6.0E-1 | 9.0E-1 |
| Glutamate | Amino acids | 0.15 (0.04) | 9.9E-4 | 2.5E-3 |  | 0.10 (0.04) | 1.9E-2 | 7.7E-2 |  | 0.00 (0.04) | 9.3E-1 | 9.8E-1 |  | -0.06 (0.05) | 1.8E-1 | 8.2E-1 |
| Glycine | Amino acids | 0.02 (0.04) | 6.3E-1 | 7.2E-1 |  | 0.02 (0.04) | 6.1E-1 | 6.6E-1 |  | -0.05 (0.04) | 2.2E-1 | 8.8E-1 |  | -0.05 (0.04) | 2.8E-1 | 9.0E-1 |
| Histidine | Amino acids | 0.02 (0.04) | 5.9E-1 | 7.2E-1 |  | 0.04 (0.04) | 3.2E-1 | 4.2E-1 |  | -0.03 (0.04) | 5.6E-1 | 8.8E-1 |  | -0.02 (0.04) | 6.9E-1 | 9.0E-1 |
| Isoleucine | Amino acids | 0.18 (0.04) | 9.2E-5 | 5.0E-4 |  | 0.12 (0.04) | 4.4E-3 | 4.9E-2 |  | 0.02 (0.04) | 6.1E-1 | 9.2E-1 |  | -0.03 (0.05) | 5.9E-1 | 9.0E-1 |
| Lysine | Amino acids | 0.17 (0.04) | 2.6E-4 | 9.8E-4 |  | 0.10 (0.04) | 1.9E-2 | 7.7E-2 |  | 0.01 (0.04) | 8.3E-1 | 9.8E-1 |  | -0.03 (0.05) | 4.5E-1 | 9.0E-1 |
| Leucine | Amino acids | 0.19 (0.04) | 2.6E-5 | 2.3E-4 |  | 0.12 (0.04) | 6.4E-3 | 4.9E-2 |  | 0.02 (0.04) | 6.3E-1 | 9.2E-1 |  | -0.04 (0.05) | 3.6E-1 | 9.0E-1 |
| Phenylalanine | Amino acids | 0.13 (0.04) | 2.4E-3 | 5.8E-3 |  | 0.10 (0.04) | 1.3E-2 | 7.2E-2 |  | 0.00 (0.04) | 9.4E-1 | 9.8E-1 |  | -0.03 (0.05) | 5.1E-1 | 9.0E-1 |
| Tyrosine | Amino acids | 0.11 (0.04) | 4.7E-2 | 8.2E-2 |  | 0.09 (0.04) | 5.8E-2 | 1.4E-1 |  | -0.05 (0.04) | 2.3E-1 | 8.8E-1 |  | -0.06  (0.04) | 1.7E-1 | 8.2E-1 |
| Valine | Amino acids | 0.12 (0.05) | 8.5E-3 | 1.9E-2 |  | 0.06 (0.04) | 1.3E-1 | 2.2E-1 |  | -0.03 (0.04) | 4.3E-1 | 8.8E-1 |  | -0.08  (0.05) | 7.2E-2 | 5.7E-1 |
| Glutamine | Amino acids | -0.02 (0.05) | 6.2E-1 | 7.2E-1 |  | 0.03 (0.04) | 5.1E-1 | 6.2E-1 |  | -0.02 (0.04) | 7.2E-1 | 9.7E-1 |  | 0.01  (0.04) | 8.2E-1 | 9.0E-1 |
| Formate | Organic acids | -0.07 (0.04) | 1.3E-1 | 1.9E-1 |  | -0.02 (0.04) | 6.1E-1 | 6.6E-1 |  | 0.07 (0.04) | 1.2E-1 | 8.8E-1 |  | 0.08  (0.04) | 5.3E-2 | 5.7E-1 |
| Acetate | Organic acids | 0.08 (0.05) | 8.3E-2 | 1.3E-1 |  | 0.08 (0.04) | 3.6E-2 | 9.9E-2 |  | 0.02 (0.04) | 6.8E-1 | 9.6E-1 |  | 0.01  (0.05) | 7.8E-1 | 9.0E-1 |
| Creatine | Organic acids | 0.11 (0.05) | 2.0E-2 | 3.9E-2 |  | 0.06 (0.04) | 1.1E-1 | 2.1E-1 |  | -0.04 (0.04) | 3.5E-1 | 8.8E-1 |  | -0.08  (0.04) | 9.1E-2 | 5.8E-1 |
| Glucose | Carbohydrates | 0.09 (0.04) | 5.3E-2 | 8.8E-2 |  | 0.04 (0.04) | 2.9E-1 | 4.1E-1 |  | 0.00 (0.04) | 9.8E-1 | 9.8E-1 |  | -0.06  (0.05) | 2.2E-1 | 8.2E-1 |
| Pyruvate | Carbohydrates | 0.16 (0.04) | 3.7E-4 | 1.3E-3 |  | 0.08 (0.04) | 3.4E-2 | 9.9E-2 |  | -0.03 (0.04) | 5.2E-1 | 8.8E-1 |  | -0.08  (0.04) | 7.5E-2 | 5.7E-1 |
| Citrate | Carbohydrates | 0.00 (0.04) | 9.7E-1 | 9.7E-1 |  | 0.04 (0.04) | 3.1E-1 | 4.2E-1 |  | -0.04 (0.04) | 4.0E-1 | 8.8E-1 |  | -0.03  (0.04) | 5.6E-1 | 9.0E-1 |
| Succinate | Carbohydrates | 0.09 (0.05) | 1.8E-2 | 3.6E-2 |  | 0.07 (0.04) | 2.7E-2 | 9.3E-2 |  | 0.00 (0.05) | 9.1E-1 | 9.8E-1 |  | -0.01  (0.04) | 7.7E-1 | 9.0E-1 |
| Fumarate | Carbohydrates | -0.05 (0.04) | 2.5E-1 | 3.1E-1 |  | -0.03 (0.04) | 4.3E-1 | 5.5E-1 |  | -0.04 (0.04) | 4.2E-1 | 8.8E-1 |  | -0.04  (0.04) | 3.8E-1 | 9.0E-1 |
| Lactate | Carbohydrates | 0.15 (0.04) | 6.7E-4 | 2.0E-3 |  | 0.06 (0.04) | 1.2E-1 | 2.2E-1 |  | -0.09 (0.04) | 5.1E-2 | 8.8E-1 |  | -0.16  (0.05) | 6.5E-4 | 2.5E-2 |
| *N*-Acetylated Glycoproteins | Glycoprotein | 0.18 (0.04) | 3.0E-5 | 2.3E-4 |  | 0.11 (0.04) | 9.6E-3 | 6.1E-2 |  | 0.03 (0.04) | 5.4E-1 | 8.8E-1 |  | -0.03  (0.05) | 4.8E-1 | 9.0E-1 |
| *O*-Acetylated Glycoproteins | Glycoprotein | 0.02 (0.05) | 6.6E-1 | 7.2E-1 |  | 0.02 (0.04) | 5.9E-1 | 6.6E-1 |  | 0.00 (0.04) | 9.4E-1 | 9.8E-1 |  | 0.00  (0.04) | 9.7E-1 | 9.8E-1 |
| Acetoacetate | Ketone | 0.20 (0.04) | 7.1E-6 | 9.6E-5 |  | 0.12 (0.04) | 4.3E-3 | 4.9E-2 |  | 0.03 (0.04) | 4.9E-1 | 8.8E-1 |  | -0.05  (0.05) | 3.2E-1 | 9.0E-1 |
| Bile Acids | Bile acid | 0.08 (0.04) | 7.7E-2 | 1.2E-1 |  | 0.02 (0.04) | 5.5E-1 | 6.5E-1 |  | 0.04 (0.04) | 3.1E-1 | 8.8E-1 |  | 0.00  (0.05) | 9.8E-1 | 9.8E-1 |
| Choline | Choline metabolites | 0.07 (0.05) | 1.4E-1 | 1.9E-1 |  | 0.06 (0.04) | 1.1E-1 | 2.1E-1 |  | 0.00 (0.04) | 9.2E-1 | 9.8E-1 |  | -0.01  (0.05) | 8.0E-1 | 9.0E-1 |
| Glycerophosphocholine | Choline metabolites | -0.01 (0.05) | 8.8E-1 | 9.3E-1 |  | 0.01 (0.04) | 9.0E-1 | 9.0E-1 |  | 0.00 (0.04) | 9.8E-1 | 9.8E-1 |  | 0.01  (0.04) | 7.6E-1 | 9.0E-1 |
| Phosphorylcholine | Choline metabolites | 0.06 (0.05) | 1.7E-1 | 2.3E-1 |  | 0.06 (0.04) | 1.6E-1 | 2.6E-1 |  | 0.00 (0.04) | 9.4E-1 | 9.8E-1 |  | -0.02  (0.05) | 7.2E-1 | 9.0E-1 |
| Hypoxanthine | Choline metabolites | 0.02 (0.04) | 6.6E-1 | 7.2E-1 |  | -0.01 (0.04) | 8.1E-1 | 8.6E-1 |  | -0.03 (0.04) | 4.6E-1 | 8.8E-1 |  | -0.05  (0.04) | 2.2E-1 | 8.2E-1 |
| Lipids (C=CC*H2*C=C) | Lipids | 0.17 (0.04) | 1.9E-4 | 7.8E-4 |  | 0.09 (0.04) | 3.6E-2 | 9.9E-2 |  | 0.06 (0.04) | 2.1E-1 | 8.8E-1 |  | -0.01  (0.05) | 8.3E-1 | 9.0E-1 |
| Lipids (C*H*=CH) | Lipids | 0.16 (0.04) | 4.7E-4 | 1.5E-3 |  | 0.08 (0.04) | 7.7E-2 | 1.7E-1 |  | 0.05 (0.04) | 2.2E-1 | 8.8E-1 |  | -0.01  (0.05) | 8.2E-1 | 9.0E-1 |
| Lipids (C*H2*C=C) | Lipids | 0.20 (0.04) | 7.6E-6 | 9.6E-5 |  | 0.12 (0.04) | 5.2E-3 | 4.9E-2 |  | 0.04 (0.04) | 3.6E-1 | 8.8E-1 |  | -0.03  (0.05) | 5.3E-1 | 9.0E-1 |
| Lipids (C*H2*CH2COO) | Lipids | 0.18 (0.04) | 4.6E-5 | 2.9E-4 |  | 0.10 (0.04) | 2.0E-2 | 7.7E-2 |  | 0.04 (0.04) | 3.3E-1 | 8.8E-1 |  | -0.03  (0.05) | 4.8E-1 | 9.0E-1 |
| Lipids (C*H2*COO) | Lipids | 0.21 (0.04) | 2.6E-6 | 9.6E-5 |  | 0.14 (0.04) | 1.0E-3 | 4.0E-2 |  | 0.03 (0.04) | 4.7E-1 | 8.8E-1 |  | -0.04  (0.05) | 3.9E-1 | 9.0E-1 |
| Lipids (R-C*H2*) | Lipids | 0.17 (0.04) | 1.8E-4 | 7.8E-4 |  | 0.08 (0.04) | 5.8E-2 | 1.4E-1 |  | 0.05 (0.04) | 2.8E-1 | 8.8E-1 |  | -0.03  (0.05) | 6.0E-1 | 9.0E-1 |
| Lipids (R-C*H3*) | Lipids | 0.15 (0.04) | 8.9E-4 | 2.4E-3 |  | 0.07 (0.04) | 8.6E-2 | 1.8E-1 |  | 0.05 (0.04) | 2.4E-1 | 8.8E-1 |  | -0.01  (0.05 | 8.7E-1 | 9.2E-1 |
| Triglycerides | Lipids | 0.11 (0.04) | 1.0E-2 | 2.1E-2 |  | 0.05 (0.04) | 2.3E-1 | 3.6E-1 |  | 0.07 (0.04) | 9.1E-2 | 8.8E-1 |  | 0.02  (0.05) | 5.9E-1 | 9.0E-1 |
| Dimethylglycine | Chemical intermediates | 0.01 (0.05) | 9.1E-1 | 9.3E-1 |  | 0.01 (0.04) | 8.6E-1 | 8.8E-1 |  | 0.03 (0.04) | 5.5E-1 | 8.8E-1 |  | 0.02  (0.04) | 6.3E-1 | 9.0E-1 |

Abbreviations: baPWV, brachial-ankle pulse wave velocity; FDR, false discovery rate; IMT, carotid intima-media thickness; SE, standard error.

Model 1 was adjusted for age and sex; Model 2 was additionally adjusted for current smoking (yes/no), physical exercise (yes/no), body mass index (continues), systolic blood pressure (continues), use of antihypertension medications (yes/no), diabetes mellitus (yes/no), and hyperlipidemia (yes/no). Data were presented in regression coefficient for per standard deviation increase in a given metabolite concentration.
