# Supplementary material for: Associations between serum metabolites and subclinical atherosclerosis in a Chinese population: the Taizhou Imaging Study
Source: Aging (Albany NY). 2020 Jul 9;12(15):15302–13. doi: 10.18632/aging.103456 (PMC7467377; doi:10.18632/aging.103456)
Supplement: Supplementary Table 4 [file aging-12-103456-s003..docx]

**Supplementary Table 4. Binary logistic regression analyses of serum metabolites and subclinical atherosclerosis.**

| Metabolites | High baPWV (baPMV >15.1 m/s) | | | | | | |  | Increased IMT (IMT >0.80 mm) | | | | | | |
| --- | --- | --- | --- | --- | --- | --- | --- | --- | --- | --- | --- | --- | --- | --- | --- |
|  | Model 1 | | |  | Model 2 | | |  | Model 1 | | |  | Model 2 | | |
|  | OR  (95% CI) | *P* value | FDR |  | OR  (95% CI) | *P* value | FDR |  | OR  (95% CI) | *P* value | FDR |  | OR  (95% CI) | *P* value | FDR |
| Alanine | 1.22  (1.01, 1.48) | 4.4E-2 | 9.8E-2 |  | 1.21  (0.96, 1.52) | 1.1E-1 | 2.6E-1 |  | 0.89  (0.73, 1.08) | 2.4E-1 | 9.5E-1 |  | 0.82  (0.67, 1.01) | 6.7E-2 | 6.4E-1 |
| Asparagine | 1.05  (0.87, 1.27) | 6.2E-1 | 7.1E-1 |  | 1.03  (0.83, 1.28) | 7.8E-1 | 8.6E-1 |  | 0.97  (0.80, 1.18) | 7.9E-1 | 9.5E-1 |  | 0.95  (0.77, 1.16) | 5.9E-1 | 9.0E-1 |
| Glutamate | 1.26  (1.04, 1.53) | 1.9E-2 | 4.8E-2 |  | 1.21  (0.96, 1.53) | 1.1E-1 | 2.6E-1 |  | 0.98  (0.81, 1.19) | 8.4E-1 | 9.5E-1 |  | 0.90  (0.72, 1.11) | 3.2E-1 | 9.0E-1 |
| Glycine | 1.02  (0.84, 1.23) | 8.6E-1 | 9.1E-1 |  | 1.02  (0.82, 1.27) | 8.6E-1 | 8.6E-1 |  | 0.91  (0.75, 1.11) | 3.5E-1 | 9.5E-1 |  | 0.92  (0.75, 1.12) | 4.0E-1 | 9.0E-1 |
| Histidine | 1.00  (0.83, 1.21) | 1.0E-0 | 1.0E-0 |  | 1.05  (0.85, 1.31) | 6.5E-1 | 8.3E-1 |  | 1.04  (0.86, 1.26) | 6.7E-1 | 9.5E-1 |  | 1.05  (0.86, 1.29) | 6.2E-1 | 9.0E-1 |
| Isoleucine | 1.34  (1.10, 1.63) | 3.8E-3 | 1.2E-2 |  | 1.28  (1.01, 1.62) | 4.4E-2 | 1.4E-1 |  | 1.03  (0.85, 1.26) | 7.5E-1 | 9.5E-1 |  | 0.97  (0.78, 1.20) | 7.7E-1 | 9.7E-1 |
| Lysine | 1.33  (1.10, 1.63) | 4.3E-3 | 1.3E-2 |  | 1.24  (0.99, 1.58) | 6.7E-2 | 2.0E-1 |  | 1.02  (0.84, 1.24) | 8.4E-1 | 9.5E-1 |  | 0.97  (0.78, 1.19) | 7.5E-1 | 9.7E-1 |
| Leucine | 1.43  (1.17, 1.75) | 4.4E-4 | 1.5E-3 |  | 1.35  (1.05, 1.73) | 1.8E-2 | 6.3E-2 |  | 1.03  (0.84, 1.25) | 7.8E-1 | 9.5E-1 |  | 0.93  (0.75, 1.16) | 5.4E-1 | 9.0E-1 |
| Phenylalanine | 1.20  (1.00, 1.45) | 5.7E-2 | 1.1E-1 |  | 1.21  (0.97, 1.52) | 9.6E-2 | 2.6E-1 |  | 1.03  (0.85, 1.25) | 7.7E-1 | 9.5E-1 |  | 0.99  (0.81, 1.22) | 9.4E-1 | 9.7E-1 |
| Tyrosine | 1.13  (0.93, 1.37) | 2.1E-1 | 3.3E-1 |  | 1.15  (0.92, 1.44) | 2.2E-1 | 3.9E-1 |  | 0.89  (0.74, 1.08) | 2.5E-1 | 9.5E-1 |  | 0.88  (0.71, 1.08) | 2.1E-1 | 9.0E-1 |
| Valine | 1.24  (1.02, 1.51) | 3.1E-2 | 7.3E-2 |  | 1.17  (0.93, 1.49) | 1.9E-1 | 3.7E-1 |  | 0.90  (0.74, 1.10) | 2.9E-1 | 9.5E-1 |  | 0.83  (0.67, 1.03) | 9.8E-2 | 7.5E-1 |
| Glutamine | 0.93  (0.76, 1.12) | 4.3E-1 | 5.7E-1 |  | 1.03  (0.83, 1.27) | 8.2E-1 | 8.6E-1 |  | 1.05  (0.86, 1.27) | 6.4E-1 | 9.5E-1 |  | 1.08  (0.88, 1.32) | 4.5E-1 | 9.0E-1 |
| Formate | 0.89  (0.73, 1.07) | 2.2E-1 | 3.3E-1 |  | 0.96  (0.77, 1.19) | 7.1E-1 | 8.6E-1 |  | 1.24  (1.02, 1.51) | 3.3E-2 | 6.5E-1 |  | 1.26  (1.03, 1.54) | 2.4E-2 | 3.1E-1 |
| Acetate | 1.10  (0.91, 1.34) | 3.2E-1 | 4.5E-1 |  | 1.16  (0.93, 1.47) | 1.9E-1 | 3.7E-1 |  | 1.13  (0.92, 1.38) | 2.4E-1 | 9.5E-1 |  | 1.11  (0.91, 1.37) | 3.1E-1 | 9.0E-1 |
| Creatine | 1.18  (0.98, 1.44) | 8.5E-2 | 1.5E-1 |  | 1.15  (0.92, 1.43) | 2.3E-1 | 4.0E-1 |  | 0.91  (0.75, 1.11) | 3.5E-1 | 9.5E-1 |  | 0.86  (0.70, 1.06) | 1.5E-1 | 8.3E-1 |
| Glucose | 1.13  (0.93, 1.36) | 2.2E-1 | 3.3E-1 |  | 1.07  (0.85, 1.35) | 5.6E-1 | 7.9E-1 |  | 1.01  (0.83, 1.22) | 9.5E-1 | 9.5E-1 |  | 0.92  (0.74, 1.14) | 4.5E-1 | 9.0E-1 |
| Pyruvate | 1.43  (1.17, 1.74) | 3.9E-4 | 1.5E-3 |  | 1.32  (1.05, 1.65) | 1.6E-2 | 6.1E-2 |  | 0.93  (0.77, 1.12) | 4.4E-1 | 9.5E-1 |  | 0.86  (0.70, 1.05) | 1.4E-1 | 8.3E-1 |
| Citrate | 1.05  (0.87, 1.27) | 5.9E-1 | 7.0E-1 |  | 1.16  (0.93, 1.45) | 1.8E-1 | 3.7E-1 |  | 0.98  (0.81, 1.19) | 8.7E-1 | 9.5E-1 |  | 1.00  (0.82, 1.21) | 9.7E-1 | 9.7E-1 |
| Succinate | 1.19  (0.98, 1.45) | 8.0E-2 | 1.5E-1 |  | 1.20  (0.96, 1.50) | 1.2E-1 | 2.7E-1 |  | 0.99  (0.81, 1.21) | 9.3E-1 | 9.5E-1 |  | 0.98  (0.80, 1.20) | 8.4E-1 | 9.7E-1 |
| Fumarate | 0.89  (0.74, 1.08) | 2.3E-1 | 3.4E-1 |  | 0.91  (0.73, 1.13) | 4.0E-1 | 5.9E-1 |  | 0.97  (0.80, 1.17) | 7.2E-1 | 9.5E-1 |  | 0.94  (0.78, 1.15) | 5.6E-1 | 9.0E-1 |
| Lactate | 1.29  (1.07, 1.57) | 9.4E-3 | 2.6E-2 |  | 1.14  (0.91, 1.44) | 2.6E-1 | 4.2E-1 |  | 0.86  (0.70, 1.04) | 1.2E-1 | 9.5E-1 |  | 0.77  (0.62, 0.95) | 1.7E-2 | 3.1E-1 |
| *N*-Acetylated Glycoproteins | 1.43  (1.18, 1.75) | 2.7E-4 | 1.2E-3 |  | 1.34  (1.06, 1.70) | 1.5E-2 | 6.1E-2 |  | 1.02  (0.84, 1.24) | 8.5E-1 | 9.5E-1 |  | 0.93  (0.75, 1.15) | 5.0E-1 | 9.0E-1 |
| *O*-Acetylated Glycoproteins | 1.00  (0.83, 1.22) | 9.6E-1 | 9.9E-1 |  | 1.02  (0.82, 1.27) | 8.4E-1 | 8.6E-1 |  | 1.08  (0.89, 1.31) | 4.5E-1 | 9.5E-1 |  | 1.08  (0.89, 1.33) | 4.3E-1 | 9.0E-1 |
| Acetoacetate | 1.59  (1.31, 1.96) | 5.6E-6 | 1.7E-4 |  | 1.53  (1.20, 1.97) | 7.8E-4 | 1.7E-2 |  | 1.04  (0.86, 1.26) | 7.1E-1 | 9.5E-1 |  | 0.91  (0.73, 1.14) | 4.2E-1 | 9.0E-1 |
| Bile Acids | 1.21  (1.00, 1.46) | 4.9E-2 | 1.0E-1 |  | 1.14  (0.91, 1.44) | 2.7E-1 | 4.2E-1 |  | 1.11  (0.91, 1.34) | 3.0E-1 | 9.5E-1 |  | 1.01  (0.82, 1.25) | 9.0E-1 | 9.7E-1 |
| Choline | 1.06  (0.87, 1.29) | 5.6E-1 | 6.9E-1 |  | 1.06  (0.84, 1.33) | 6.4E-1 | 8.3E-1 |  | 0.97  (0.80, 1.18) | 7.7E-1 | 9.5E-1 |  | 0.92  (0.75, 1.13) | 4.3E-1 | 9.0E-1 |
| Glycerophosphocholine | 0.92  (0.76, 1.11) | 3.7E-1 | 5.0E-1 |  | 0.89  (0.71, 1.11) | 3.0E-1 | 4.6E-1 |  | 1.01  (0.83, 1.22) | 9.3E-1 | 9.5E-1 |  | 1.01  (0.82, 1.24) | 9.4E-1 | 9.7E-1 |
| Phosphorylcholine | 1.05  (0.86, 1.27) | 6.5E-1 | 7.3E-1 |  | 1.03  (0.82, 1.29) | 7.9E-1 | 8.6E-1 |  | 0.99  (0.81, 1.20) | 9.1E-1 | 9.5E-1 |  | 0.93  (0.76, 1.15) | 5.1E-1 | 9.0E-1 |
| Hypoxanthine | 1.06  (0.88, 1.28) | 5.4E-1 | 6.9E-1 |  | 1.03  (0.83, 1.27) | 8.2E-1 | 8.6E-1 |  | 0.81  (0.66, 0.98) | 3.4E-2 | 6.5E-1 |  | 0.76  (0.62, 0.93) | 8.2E-3 | 3.1E-1 |
| Lipids (C=CC*H2*C=C) | 1.47  (1.21, 1.79) | 1.3E-4 | 8.5E-4 |  | 1.39  (1.09, 1.78) | 8.8E-3 | 5.6E-2 |  | 1.08  (0.89, 1.31) | 4.5E-1 | 9.5E-1 |  | 0.97  (0.77, 1.2) | 7.6E-1 | 9.7E-1 |
| Lipids (C*H*=CH) | 1.44  (1.19, 1.76) | 2.5E-4 | 1.2E-3 |  | 1.36  (1.07, 1.74) | 1.4E-2 | 6.1E-2 |  | 1.09  (0.90, 1.33) | 3.7E-1 | 9.5E-1 |  | 0.98  (0.79, 1.23) | 8.8E-1 | 9.7E-1 |
| Lipids (C*H2*C=C) | 1.55  (1.28, 1.90) | 1.4E-5 | 1.7E-4 |  | 1.49  (1.17, 1.91) | 1.5E-3 | 2.0E-2 |  | 1.03  (0.85, 1.26) | 7.4E-1 | 9.5E-1 |  | 0.92  (0.74, 1.15) | 4.7E-1 | 9.0E-1 |
| Lipids (C*H2*CH2COO) | 1.53  (1.26, 1.87) | 2.5E-5 | 2.4E-4 |  | 1.47  (1.15, 1.9) | 2.4E-3 | 2.3E-2 |  | 1.03  (0.85, 1.26) | 7.3E-1 | 9.5E-1 |  | 0.91  (0.72, 1.13) | 3.9E-1 | 9.0E-1 |
| Lipids (C*H2*COO) | 1.56  (1.28, 1.9) | 1.3E-5 | 1.7E-4 |  | 1.52  (1.19, 1.95) | 9.0E-4 | 1.7E-2 |  | 1.01  (0.83, 1.22) | 9.5E-1 | 9.5E-1 |  | 0.89  (0.71, 1.11) | 2.9E-1 | 9.0E-1 |
| Lipids (R-C*H2*) | 1.51  (1.25, 1.85) | 4.0E-5 | 3.0E-4 |  | 1.44  (1.13, 1.86) | 4.0E-3 | 3.0E-2 |  | 1.06  (0.87, 1.29) | 5.4E-1 | 9.5E-1 |  | 0.94  (0.75, 1.18) | 6.0E-1 | 9.0E-1 |
| Lipids (R-C*H3*) | 1.45  (1.19, 1.77) | 2.3E-4 | 1.2E-3 |  | 1.37  (1.08, 1.75) | 1.1E-2 | 6.1E-2 |  | 1.07  (0.88, 1.31) | 4.7E-1 | 9.5E-1 |  | 0.97  (0.78, 1.21) | 7.8E-1 | 9.7E-1 |
| Triglycerides | 1.16  (0.96, 1.41) | 1.2E-1 | 2.0E-1 |  | 1.06  (0.84, 1.33) | 6.4E-1 | 8.3E-1 |  | 1.06  (0.87, 1.29) | 5.7E-1 | 9.5E-1 |  | 0.97  (0.79, 1.20) | 8.0E-1 | 9.7E-1 |
| Dimethylglycine | 0.98  (0.81, 1.18) | 8.0E-1 | 8.6E-1 |  | 0.97  (0.78, 1.21) | 7.8E-1 | 8.6E-1 |  | 1.03  (0.85, 1.25) | 7.6E-1 | 9.5E-1 |  | 1.02  (0.83, 1.24) | 8.7E-1 | 9.7E-1 |

Abbreviations: baPWV, brachial-ankle pulse wave velocity; CI, confidence interval; FDR, false discovery rate; IMT, carotid intima-media thickness; OR, odds ratio.

Model 1 was adjusted for age and sex; Model 2 was additionally adjusted for current smoking (yes/no), physical exercise (yes/no), body mass index (continues), systolic blood pressure (continues), use of antihypertension medications (yes/no), diabetes mellitus (yes/no), and hyperlipidemia (yes/no). Data were presented in OR for per standard deviation increase in a given metabolite concentration.
